# AGR2 promotes tumor progression by regulating macrophage polarization via the CD98hc-xCT/p-ERK pathway

**DOI:** 10.3389/fimmu.2026.1774238

**Published:** 2026-02-26

**Authors:** Naming Wu, Liang Zhao, Shan Jiang

**Affiliations:** Department of Dermatology, Renmin Hospital of Wuhan University, Wuhan, Hubei, China

**Keywords:** AGR2, CD98hc−xCT, macrophage polarization, p−ERK/ERK pathway, tumor microenvironment

## Abstract

**Introduction:**

Anterior gradient 2 (AGR2) contributes to tumorigenesis, yet its function within the tumor microenvironment (TME) and in macrophage polarization remains unclear. This study assessed the prognostic significance of AGR2 and investigated its mechanism in cancer progression.

**Methods:**

We used TCGA for pan-cancer AGR2 expression and survival analysis, examined macrophage infiltration in clinical specimens, and performed in vitro experiments with recombinant AGR2 (rAGR2) to assess macrophage polarization. We verified AGR2’s interaction with the CD98hc-xCT receptor complex and explored related mechanisms via CD98hc knockdown. In vivo experiments were conducted in B16-F10 melanoma and Lewis lung carcinoma (LLC) models using flow cytometry.

**Results:**

Pan−cancer analyses showed that elevated AGR2 expression correlates with poor prognosis in multiple cancers and is associated with reduced immune infiltration. AGR2 is predominantly expressed in CD163+ M2−like tumor−associated macrophages (TAMs), with levels rising alongside tumor stage. In vitro, rAGR2 promoted M2 polarization while inhibiting M1 polarization of macrophages, and enhanced the pro−tumorigenic effects of M2−conditioned medium on cancer cell motility and proliferation. Mechanistically, AGR2 binds to the CD98hc−xCT receptor complex, activating the ERK pathway, an effect abrogated by CD98hc knockdown. In vivo, rAGR2 accelerated tumor growth in melanoma and lung cancer models, accompanied by increased TAM accumulation, a shift toward M2 polarization, and suppressed T-cell function.

**Discussion:**

AGR2 drives tumor progression by reprogramming TAMs toward an M2 phenotype and attenuating T−cell function via the CD98hc−xCT/p-ERK pathway, highlighting its potential as both a prognostic marker and a therapeutic target.

## 1 Introduction

Cancer progression is a complex multistep process orchestrated by both tumor cells and the surrounding tumor microenvironment (TME) (1, 2). The TME, consisting of stromal cells, immune cells, and extracellular matrix components, plays a pivotal role in regulating tumor growth, invasion, metastasis, and therapeutic response (3, 4). Among the diverse immune cells in the TME, tumor-associated macrophages (TAMs) are the most abundant population, exhibiting high plasticity that allows them to polarize into distinct functional phenotypes (5–7). Classically activated M1 macrophages secrete pro-inflammatory cytokines (e.g., IFN-γ and TNF-α) and exert anti-tumor effects, while alternatively activated M2 macrophages produce anti-inflammatory factors and promote tumor progression by supporting angiogenesis, immune suppression, and tumor cell proliferation (8–10). The balance between M1 and M2 polarization of TAMs is thus a critical determinant of cancer outcomes, making the regulatory mechanisms of this process a key focus in oncology research (11, 12). Consistently, increased TAM infiltration correlates with poor prognosis in various solid tumors, as validated in both preclinical animal models and clinical human cancer cohorts (13, 14). Targeting TAMs—by inhibiting M2 polarization, depleting their population, or reprogramming them toward a proinflammatory M1 state—has emerged as a promising therapeutic strategy to inhibit tumor growth and metastasis, with several approaches already being applied in clinical settings (15–18).

Anterior gradient 2 (AGR2), a homolog of the Xenopus XAG2 protein, is overexpressed and secreted by cancer cells into the extracellular matrix, where it contributes to TME formation (19–21). AGR2 has been implicated in various biological processes, including cell migration, ERK signaling regulation, cellular transformation, and cell adhesion, making it a potential drug target and biomarker for circulating tumor cell detection (22–24). Elevated AGR2 levels have been observed in numerous cancers, including breast, prostate, lung, pancreatic, and colorectal cancers, as well as in hepatocellular carcinoma (fibrolamellar variant) and cholangiocarcinoma (23, 25, 26). Notably, AGR2 overexpression is associated with poor prognosis in breast cancer patients (27). However, while AGR2’s cell-autonomous roles in tumor cells are increasingly recognized, its non-cell-autonomous functions in modulating the TME—particularly its impact on TAM polarization—remain largely undefined. This knowledge gap limits our comprehensive understanding of AGR2’s role in cancer progression and hinders the development of targeted therapeutic strategies.

While AGR2’s cell-autonomous roles in tumor cells are recognized, its non-cell-autonomous functions in the TME, particularly concerning immune cell modulation, are poorly understood. Our preliminary pan-cancer analysis revealed that high AGR2 expression correlates with poor prognosis and a TME characterized by reduced immune infiltration, suggesting an immunosuppressive role. Critically, subsequent immunohistochemical analysis of tumor tissues indicated that AGR2 is predominantly expressed in CD163^+^ TAMs, a population canonically associated with the immunosuppressive M2 phenotype. Based on (1) the established central role of M2-polarized TAMs in fostering tumor progression and immune evasion, and (2) our specific observation of AGR2 enrichment in CD163^+^ TAMs, we hypothesized that AGR2 contributes to tumor progression by actively reprogramming macrophages towards an M2-polarized state. This study aims to test this hypothesis and elucidate the underlying molecular mechanism.

CD98 heavy chain (CD98hc), together with its light chain xCT, forms a heterodimeric receptor complex that mediates the uptake of neutral amino acids and regulates cellular redox homeostasis (28, 29). A recent study identified a novel crosstalk between tumor-associated neutrophils (TANs) and colorectal cancer (CRC) cells mediated by the secreted AGR2–CD98hc–xCT axis, which promotes cancer metastasis and influences patient outcomes (19). The ERK pathway, a key downstream signaling cascade of the CD98hc-xCT complex, is well-documented to regulate macrophage functional phenotypes and tumor cell survival (30). However, whether AGR2 interacts with the CD98hc-xCT complex to modulate ERK signaling and thereby regulate TAM polarization and TME remodeling remains unaddressed.

In this study, we aimed to clarify the prognostic value of AGR2 and its underlying mechanism of action in cancer progression. Pan-cancer analysis revealed that AGR2 was upregulated in 20 cancer types and correlated with poor overall survival in liver hepatocellular carcinoma (LIHC), lung adenocarcinoma (LUAD), and esophageal carcinoma (ESCA). High AGR2 expression was associated with increased tumor purity and reduced stromal/immune cell infiltration. Immunohistochemical analysis demonstrated that AGR2 was predominantly expressed in CD163-positive M2-like TAMs, with its expression increasing with tumor stage. *In vitro*, recombinant AGR2 (rAGR2) promoted interleukin-4 (IL-4)-induced M2 polarization and suppressed lipopolysaccharide (LPS)-induced M1 polarization of bone marrow-derived macrophages (BMDMs). M2-conditioned medium (M2-CM)-induced migration, invasion, and proliferation of B16-F10 cells were further enhanced by rAGR2. Mechanistically, AGR2 bound to the CD98hc-xCT receptor complex and activated the p-ERK/ERK signaling pathway, and CD98hc knockdown abrogated AGR2-mediated ERK activation. *In vivo*, rAGR2 treatment significantly promoted tumor growth in both B16-F10 and Lewis lung carcinoma (LLC) xenograft models, which was accompanied by increased TAM accumulation, enhanced M2 polarization, reduced M1 polarization, and suppressed IFN-γ/TNF-α secretion by CD4^+^ and CD8^+^ T cells. In summary, AGR2 serves as a prognostic biomarker and promotes cancer progression by regulating macrophage polarization and suppressing anti-tumor T cell function via the CD98hc-xCT/p-ERK pathway. Targeting AGR2 may provide a novel therapeutic strategy for cancer treatment.

## 2 Materials and methods

### 2.1 Human samples

Tumor tissue samples were obtained from archived specimens in the Department of Pathology, Renmin Hospital of Wuhan University, between January 2024 and June 2025. These samples consisted of de-identified residual clinical specimens archived after completion of routine diagnostic procedures. Paraffin-embedded sections of these samples were prepared for immunofluorescence staining to analyze AGR2 expression and macrophage polarization patterns.

### 2.2 Mice

Female C57BL/6 wild-type mice (6–8 weeks old) were obtained from the laboratory animal center of the First clinical college of Wuhan University. All mice were housed under specific pathogen-free (SPF) conditions. For anesthesia, isoflurane was used: 3-5% in oxygen for induction, 1-2% for maintenance during procedures. Vital signs (respiratory and heart rate) were monitored. Euthanasia post-experiment utilized CO_2_ inhalation per AVMA guidelines: mice were placed in a closed chamber with CO_2_ at 30-70% chamber volume displacement/minute. They remained 5+ minutes post-respiratory arrest; cervical dislocation was used if needed to confirm death. The animal study was conducted from January 2024 to June 2025. All procedures and reporting of this animal study comply with the ARRIVE (Animal Research: Reporting of *In Vivo* Experiments) guidelines to ensure the transparency and reproducibility of the research.

### 2.3 Cell lines

Mouse B16-F10 and LLC cells were obtained from the American Type Culture Collection (ATCC). Both cell lines were cultured in Dulbecco’s Modified Eagle Medium (DMEM) supplemented with 10% fetal bovine serum (FBS, Gibco, CAT# 10099141C) and 100 U/mL penicillin-streptomycin (Gibco, CAT# 15140-122). All cell lines were tested for mycoplasma contamination using the MycoAlert Mycoplasma Detection Kit (Lonza) upon receipt and on a monthly basis during culture, with all results confirming they were contamination-free.

### 2.4 Analysis of AGR2 expression in pan-cancer datasets

Unified and standardized pan-cancer datasets were downloaded from the UCSC database (https://xenabrowser.net/), including data from The Cancer Genome Atlas (TCGA), Therapeutically Applicable Research to Generate Effective Treatments (TARGET), and Genotype-Tissue Expression (GTEx) projects. A total of 19,131 samples and 60,499 gene expression profiles were included. Expression data for the AGR2 gene (ENSG00000116254) were extracted for each sample.

### 2.5 Survival prognosis analysis

The association between AGR2 expression and overall survival (OS) in pan-cancer was analyzed using the Kaplan-Meier Plotter (https://kmplot.com/analysis/) (31). A *p*-value < 0.05 was considered statistically significant.

### 2.6 Correlation between AGR2 expression and the TME across different types of cancers

Immune cell infiltration scores were downloaded and analyzed from the TCGA ImmuCellAI Database (https://guolab.wchscu.cn/ImmuCellAI/#!/) (32, 33) and the TIMER2 database (http://timer.cistrome.org/) (34–36). Patients within each tumor type in the TCGA datasets were divided into two groups based on median AGR2 expression levels to compare immune cell infiltration. The abundance of specific immune cell populations in tumor tissues (n = 44) was estimated using CIBERSORT, which analyzes gene expression data to infer cell type proportions.

### 2.7 Immunofluorescence assay

For immunofluorescence staining of tumor tissues, 4 μm paraffin-embedded tissue sections were deparaffinized in xylene and rehydrated through an ethanol gradient (90%/80%/70%). Antigen retrieval was performed by boiling sections in enhanced citrate antigen retrieval solution. Non-specific binding was blocked with 5% bovine serum albumin (BSA). Tissues were permeabilized with 0.5% Triton X-100 for 30 minutes at room temperature, followed by incubation with primary and secondary antibodies. Antibody details and dilutions are provided in **Supplementary Table 1**. For the quantification of AGR2 expression in macrophages, five random high-power fields (200× magnification) per tissue section were imaged using a laser-scanning confocal microscope. Quantitative analysis was performed using ImageJ. Single-channel images for DAPI (nuclei), AGR2, and the macrophage markers (CD68 or CD163) were analyzed separately. Individual cells were segmented based on the DAPI signal to define nuclear boundaries. A cellular region of interest (ROI) was generated for each nucleus by applying a fixed cytoplasmic expansion. A binary mask for macrophage-positive cells was created by applying an automated threshold (Otsu’s method) to the CD68 or CD163 channel. Only cells positive for the macrophage marker were included in the analysis. The mean fluorescence intensity (MFI) of AGR2 was measured within the macrophage-positive ROI of each cell. The relative AGR2 expression level for each sample was calculated as the average MFI per cell and normalized to the control group (set as 1).

### 2.8 Macrophage preparation and stimulation

BMDMs were isolated as previously described. Briefly, femurs and tibias from 6–8-week-old female C57BL/6 mice were flushed with sterile phosphate-buffered saline (PBS). Bone marrow cells were plated in 24-well plates (Costar) and cultured in DMEM supplemented with 10% FBS and 20 ng/mL mouse macrophage colony-stimulating factor (M-CSF, R&D Systems, CAT# 416-ML-050). After 8 days, adherent cells were washed with PBS and stimulated with 20 ng/mL IL-4 (PeproTech, CAT# 214-14-20) for 24 hours or 100 ng/mL LPS (InvivoGen, CAT# tlrl-3pelps) for 6 hours. This protocol yielded 2–6 × 10^7^ macrophages per mouse (two femurs and two tibias), as confirmed by FACS analysis of F4/80 expression.

### 2.9 Real-time quantitative PCR

Total RNA was extracted using TRIzol reagent (Invitrogen, CAT# 15596018) according to the manufacturer’s protocol. cDNA was synthesized using the RevertAid First Strand cDNA Synthesis Kit (Thermo Fisher, CAT# K1622). RT-PCR was performed in triplicate using SYBR Green Master Mix (Roche, CAT# 06924204001). Beta-actin was used as an internal reference for mRNA normalization. Relative mRNA expression was calculated using the 2^−ΔΔCT^ method. Primer sequences are listed in **Supplementary Table 2**.

### 2.10 Western blot assay

Cell pellets were lysed in lysis buffer containing protease and RNase inhibitors. Protein concentrations were quantified using the BCA assay. Proteins were denatured in 5x loading buffer, separated by 10% SDS-PAGE, and transferred to polyvinylidene fluoride (PVDF) membranes. Membranes were blocked with 5% BSA for 2 hours at room temperature, followed by overnight incubation at 4 °C with primary antibodies against ERK (Cell Signaling Technology, CAT# 4695S), p-ERK (Cell Signaling Technology, CAT# 4370S), iNOS (Santa Cruz, CAT# sc-650), β-actin (Proteintech, CAT# 66009-1-Ig), and Arg1 (Proteintech, CAT# 16001-1-AP). After washing with TBST, membranes were incubated with HRP-conjugated secondary antibodies for 1 hour at room temperature. Protein bands were visualized using a Bio-Rad imaging system and analyzed with Image Lab software (version 6.0.1).

### 2.11 Enzyme-linked immunosorbent assay

Concentrations of IL-6 (Biolegend, CAT# 431304), IL-1β (Biolegend, CAT# 432604), and TNF-α (Biolegend, CAT# 430904) in cell culture supernatants were measured using sandwich ELISA kits following the manufacturer’s instructions with standardization. Briefly, 96-well plates pre-coated with capture antibodies were incubated at 4 °C overnight. After washing with PBST (0.05% Tween-20), plates were blocked with 1% BSA at room temperature (RT) for 2 h. Cell supernatants (100 μL/well) and standards were added, followed by incubation at RT for 2 h. Plates were washed, then incubated with detection antibodies at RT for 1 h, and subsequently with HRP-conjugated streptavidin at RT for 30 min in the dark. After washing, the TMB substrate was added and incubated at RT for 10–15 min; the reaction was stopped with 2 M H_2_SO_4_. Absorbance was read at 450 nm (reference wavelength 570 nm), and cytokine concentrations were calculated via the standard curve.

### 2.12 Animal model

Female C57BL/6J mice (6–8 weeks old) were randomly divided into two groups. For the subcutaneous tumor model, 3 × 10^5^ B16-F10 or LLC cells resuspended in cold PBS were injected into the right flank of each mouse. rAGR2 (1 μg/g body weight) or PBS (control) was administered intraperitoneally every 2 days for a total of 17 or 15 days. Tumor length and width were measured with calipers, and tumor volume was calculated using the formula: tumor volume = length × width × width/2. At the endpoint, mice were euthanized.

### 2.13 Tumor-infiltrating immune cell isolation and analysis

Tumors were minced and incubated in RPMI medium supplemented with 320 μg/mL collagenase IV (Sigma-Aldrich, CAT# C5138), 500 μg/mL hyaluronidase type V (Sigma-Aldrich, CAT# H3506), and 5 μg/mL deoxyribonuclease I (Sigma-Aldrich, CAT# D4902) for 30 minutes at 37 °C. Single-cell suspensions were obtained by filtering through a 70 μm cell strainer (Corning), and immune cells were enriched via gradient centrifugation using lymphocyte separation medium (STEMERY, CAT# RC-001) at 524 × g for 30 minutes at 25 °C. Red blood cells were lysed with Tris-buffered ammonium chloride buffer to obtain tumor-infiltrating immune cells (TIIs). For surface marker staining, TIIs were pre-incubated with an anti-CD16/32 monoclonal antibody (Fc block, BioLegend, CAT# 101320) to block nonspecific binding, followed by incubation with fluorochrome-conjugated antibodies against CD45 (BioLegend, CAT# 157214), CD11b (BioLegend, CAT# 101245), F4/80 (BioLegend, CAT# 123132), CD206 (BioLegend, CAT# 141706), CD80 (BioLegend, CAT# 104711), and CD86 (BioLegend, CAT# 105012) according to the manufacturer’s protocol. For intracellular cytokine detection (IFN-γ and TNF-α), TIIs were stimulated for 4 hours at 37 °C with 5% CO_2_ in RPMI medium containing 10% FBS, using a cell stimulation cocktail supplemented with protein transport inhibitors: 20 ng/mL phorbol 12-myristate 13-acetate (PMA; Sigma-Aldrich), 1 μg/mL ionomycin (Sigma-Aldrich), 1:1000 brefeldin A (eBioscience), and 1:1000 monensin (eBioscience). After stimulation, cells were permeabilized and fixed using the Cytofix/Cytoperm W/Golgi Stop Kit (BD Pharmingen) at a 1:3 dilution for 30 minutes at 4 °C. Subsequently, cells were stained with fluorochrome-conjugated antibodies against CD3 (BioLegend, CAT# 100204), CD4 (BioLegend, CAT# 100430), CD8a (BioLegend, CAT# 100743), IFN-γ (BioLegend, CAT# 505832), and TNF-α (BioLegend, CAT# 506358) for 1 hour at 4 °C. All stained cells were analyzed using a FACS Calibur cytometer (BD Biosciences) and FlowJo software.

### 2.14 Wound healing assay

B16-F10 cells (4 × 10^5^ cells/well) were cultured in 6-well plates until confluent. A scratch was made using a pipette tip, and dislodged cells were washed away with PBS. Cells were incubated in M2-CM with or without 50 ng/mL or 100 ng/mL AGR2 (ProSpec, CAT# pro-2395). After 12 hours, cell migration was observed and photographed using a phase-contrast microscope. Three random fields along the scratch line were analyzed using ImageJ software.

### 2.15 Transmigration assay

M2-CM containing 50 ng/mL or 100 ng/mL AGR2 was added to the lower chamber of a 24-well plate. B16-F10 cells (2 × 10^3^) were seeded in the upper chamber. After 72 hours, cells that migrated through the membrane were stained and counted.

### 2.16 Invasion assay

Invasion assays were performed using 24-well Transwell inserts with 8 μm pores (Corning). B16-F10 cells (2 × 10^4^) were seeded in the upper chamber with M2-CM and 50 ng/mL or 100 ng/mL AGR2. After 72 hours, non-invading cells on the upper surface were removed. Invading cells on the lower surface were fixed with 4% paraformaldehyde, stained with crystal violet, and counted under a microscope at 200× magnification.

### 2.17 shRNA-mediated CD98hc silencing in B16-F10 cells

Mouse CD98hc-targeting shRNA and non-targeting negative control (NC) shRNA were purchased from Shanghai Shenggong. The target sequence for CD98hc-shRNA was 5’-GAGCCGAGAAGAAUGGUCUGGUGAA-3’, and the NC-shRNA sequence was 5’-UUCUCCGAACGUGUCACGU-3’ (scrambled sequence with no homology to mouse genes). Recombinant lentiviral vectors (pLKO.1 backbone) containing CD98hc-shRNA or NC-shRNA were co-transfected with packaging plasmids (pMDL: VSVG: REV = 5:3:2) into HEK293T cells using Lipofectamine 2000. Lentiviral supernatants were collected at 24 and 48 h post-transfection, filtered through a 0.45 μm membrane, and stored at -80 °C. B16-F10 cells were seeded in 6-well plates (30-50% confluency) and infected with lentiviral supernatants supplemented with 5 μg/mL polybrene. After 16 h, the medium was replaced with fresh complete medium. Stable cell lines were selected with 2 μg/mL puromycin for 7–10 days. Silencing efficiency was verified by RT-PCR and cell lines with ≥70% reduction in CD98hc expression (vs. NC-shRNA group) were used for subsequent experiments.

### 2.18 Statistical analysis

Data are presented as mean ± standard error of the mean (SEM). Statistical analyses were performed using GraphPad Prism 8 (GraphPad Software). Differences were assessed using a two-tailed Student’s *t*-test or one-way/two-way ANOVA, as appropriate. Significance levels were set at *P≤ 0.05, **P ≤ 0.01, and ***P ≤ 0.001.

## 3 Results

### 3.1 Increased AGR2 levels predict poor prognosis in pan-cancer analysis

**Figure 1:**
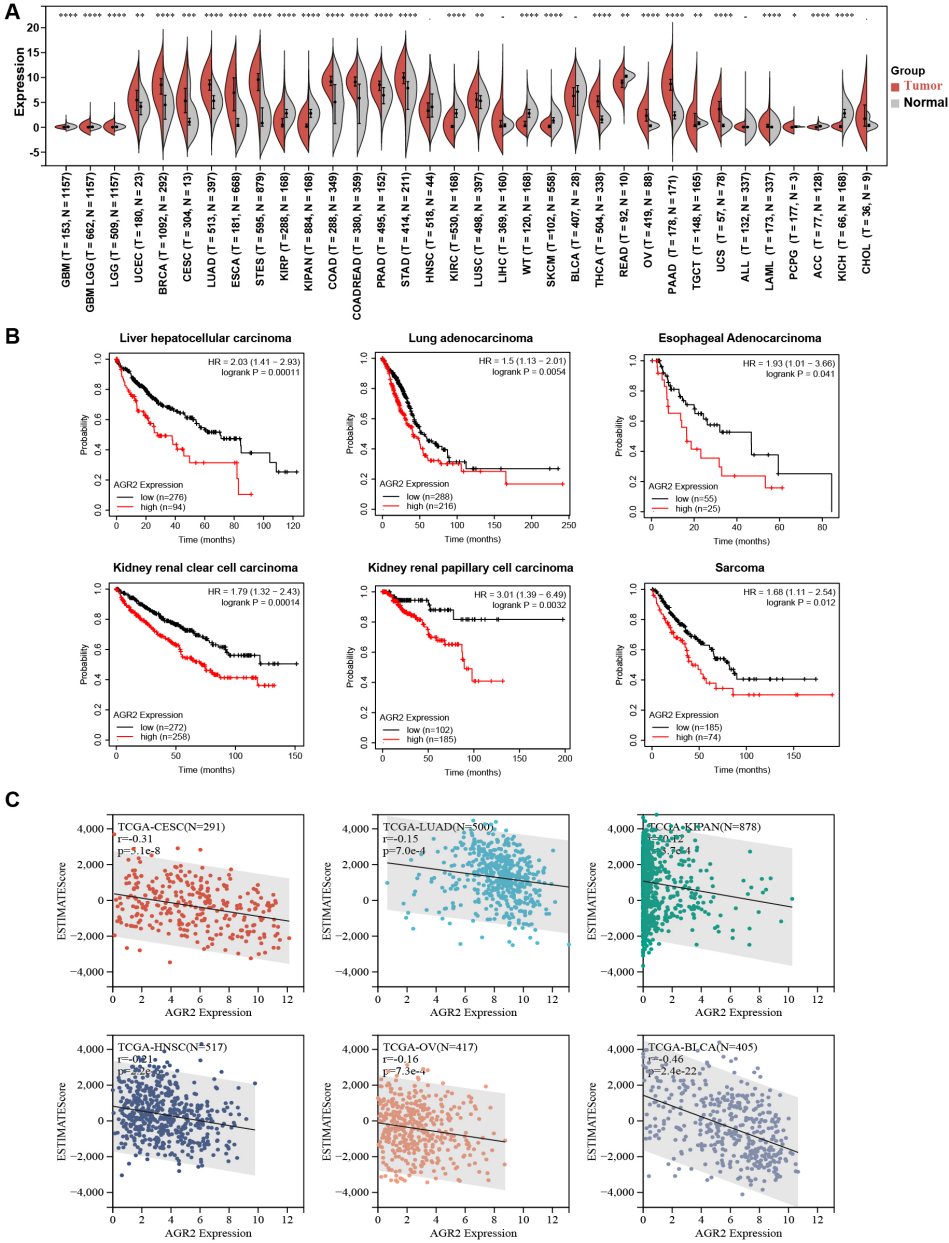
Increased AGR2 levels predict poor prognosis in pan-cancer. Abbreviations for cancer types: GBM, glioblastoma multiforme; GBMLGG, glioma; LGG, brain lower grade glioma; UCEC, uterine corpus endometrial carcinoma; BRCA, breast invasive carcinoma; CESC, cervical squamous cell carcinoma and endocervical adenocarcinoma; LUAD, lung adenocarcinoma; ESCA, esophageal carcinoma; STES, stomach and esophageal carcinoma; KIRP, kidney renal papillary cell carcinoma; KIPAN, pan-kidney cohort (KICH + KIRC + KIRP); COAD, colon adenocarcinoma; COADREAD, colon adenocarcinoma/rectum adenocarcinoma esophageal carcinoma; PRAD, prostate adenocarcinoma; STAD, stomach adenocarcinoma; HNSC, head and neck squamous cell carcinoma; KIRC, kidney renal clear cell carcinoma; LUSC, lung squamous cell carcinoma; LIHC, liver hepatocellular carcinoma; WT, Wilms tumor; SKCM, skin cutaneous melanoma; BLCA, bladder urothelial carcinoma; THCA, thyroid carcinoma; READ, rectum adenocarcinoma; OV, ovarian serous cystadenocarcinoma; PAAD, pancreatic adenocarcinoma; TGCT, testicular germ cell tumor; UCS, uterine carcinosarcoma; ALL, acute lymphoblastic leukemia; LAML, acute myeloid leukemia; PCPG, pheochromocytoma and paraganglioma; ACC, adrenocortical carcinoma; KICH, kidney chromophobe; CHOL, cholangiocarcinoma. **(A)**. Kaplan-Meier analysis of overall survival (OS) curves for liver hepatocellular carcinoma (LIHC), lung adenocarcinoma (LUAD), esophageal adenocarcinoma (ESCA), kidney renal clear cell carcinoma (KIRC), kidney renal papillary cell carcinoma (KIRP), and sarcoma patient datasets from TCGA. **(B)**. Negative correlation between AGR2 expression and immune scores in CESC, LUAD, KIPAN, HNSC, OV, and BLCA. **(C)**. *P < 0.05, **P < 0.01, ***P < 0.001, and ****P < 0.0001.

To explore the potential oncogenic role of AGR2, we compared its expression levels across 34 different cancer types and their corresponding normal tissues using data from the TCGA dataset. Our analysis revealed that AGR2 expression was significantly upregulated in tumor tissues compared to matched non-tumor tissues in 20 cancer types, including GBMLGG, LGG, UCEC, BRCA, CESC, LUAD, ESCA, STES, COAD, COADREAD, PRAD, STAD, LIHC, THCA, OV, PAAD, TGCT, UCS, PCPG, and CHOL. In contrast, lower AGR2 expression was observed in 10 tumor types: GBM, KIRP, KIPAN, HNSC, KIRC, WT, SKCM, READ, ACC, and KICH. No significant differences in AGR2 expression were detected in four cancer types: LUSC, BLCA, ALL, and LAML (**Figure 1A**). These findings suggest that AGR2 may function as an oncogene in a wide range of cancers.

Given its potential role in tumorigenesis, we further investigated the prognostic value of AGR2. Kaplan-Meier survival analysis was performed using data from the TCGA database to assess the relationship between AGR2 expression and patient outcomes. In LIHC, higher AGR2 expression was significantly associated with shorter overall survival (**Figure 1B**). Similar trends were observed in LUAD and ESCA, where elevated AGR2 levels correlated with poorer prognosis (**Figure 1B**). To further understand the impact of AGR2 on the tumor microenvironment, we employed the ESTIMATE algorithm to calculate the ESTIMATEScore, which reflects the combined abundance of stromal and immune components in tumors, and derived tumor purity as 1 minus ESTIMATEScore. Analysis of clinical data from the TCGA dataset revealed a significant negative correlation between AGR2 expression and ESTIMATEScore (**Figure 1C**). Specifically, AGR2-high tumors exhibited lower ESTIMATEScores, indicating reduced infiltration of stromal and immune cells, and correspondingly higher tumor purity. In summary, these findings demonstrate that upregulation of AGR2 is positively correlated with poor prognosis across multiple cancer types and is linked to increased tumor purity and decreased stromal/immune cell infiltration. These results underscore the potential of AGR2 as a prognostic biomarker and highlight its role in remodeling the tumor microenvironment.

### 3.2 Elevated AGR2 expression in macrophages correlates with tumor malignancy

**Figure 2:**
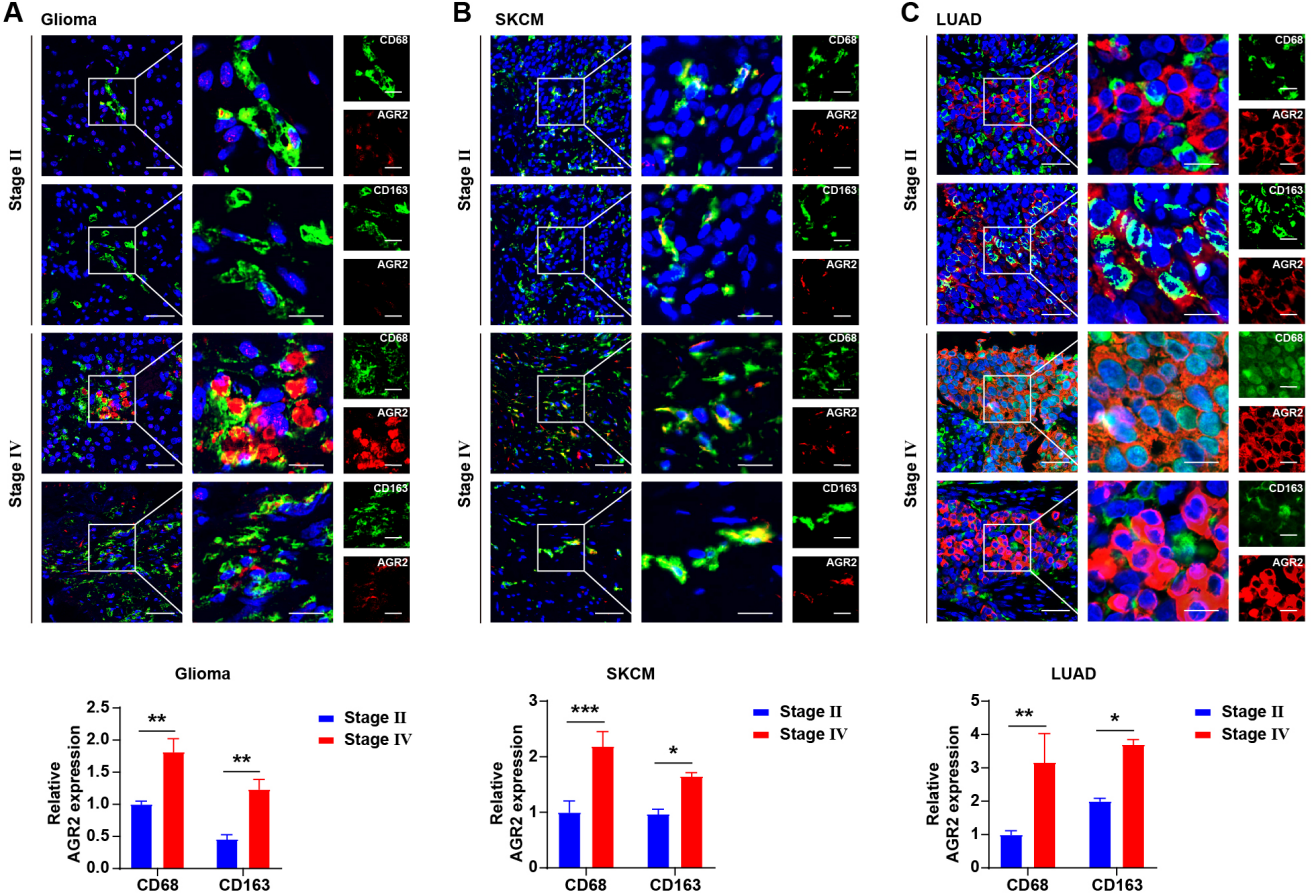
Elevated AGR2 expression in macrophages correlates with tumor malignancy. **(A–C)** Immunofluorescence staining for CD68, AGR2, and CD163 in glioma **(A)**, SKCM **(B)**, and LUAD **(C)** samples. Dual fluorescence staining was performed using anti-human CD68 (Alexa 488-Green), CD163 (Alexa 488-Green), and AGR2 (Alexa 594-Red) antibodies to co-localize CD68/AGR2 or CD163/AGR2. Nuclei were counterstained with DAPI. Scale bars: 50 μm (left panel), 20 μm (all other panels). The relative expression levels of AGR2 in CD68^+^ and CD163^+^ cells are statistically analyzed and presented below the corresponding positions. *P < 0.05, **P < 0.01, and ***P < 0.001.

To investigate the relationship between AGR2 expression and clinicopathological features of cancer, we analyzed AGR2 levels in tumor tissues from patients at different pathological stages. Our findings revealed that AGR2 expression progressively increased with advancing tumor stages in multiple cancer types, including glioma (**Figure 2A**), SKCM (**Figure 2B**), and LUAD (**Figure 2C**). Notably, immunohistochemical analysis demonstrated that AGR2 co-localized with the macrophage marker CD68, indicating its expression in macrophages. Further subtyping of macrophages revealed that AGR2 was predominantly co-localized with CD163-positive TAMs, suggesting a potential role for AGR2 in modulating TAM function within the TME. Quantitative analysis showed that AGR2 expression in TAMs was significantly higher in stage IV glioma patients compared to stage II patients. Similar trends were observed in SKCM and LUAD patients, where AGR2 levels in TAMs increased with tumor progression (**Figures 2A–C**). In summary, these results demonstrate that upregulation of AGR2 in macrophages within the TME is positively associated with tumor progression and malignancy. These findings—showing that AGR2 is specifically enriched in CD163^+^ M2-like TAMs and its expression escalates with tumor stage—led us to hypothesize that AGR2 plays a functional role in regulating macrophage polarization towards the pro-tumorigenic M2 phenotype.

### 3.3 AGR2 promotes IL-4-induced M2 polarization of macrophages *in vitro*

**Figure 3:**
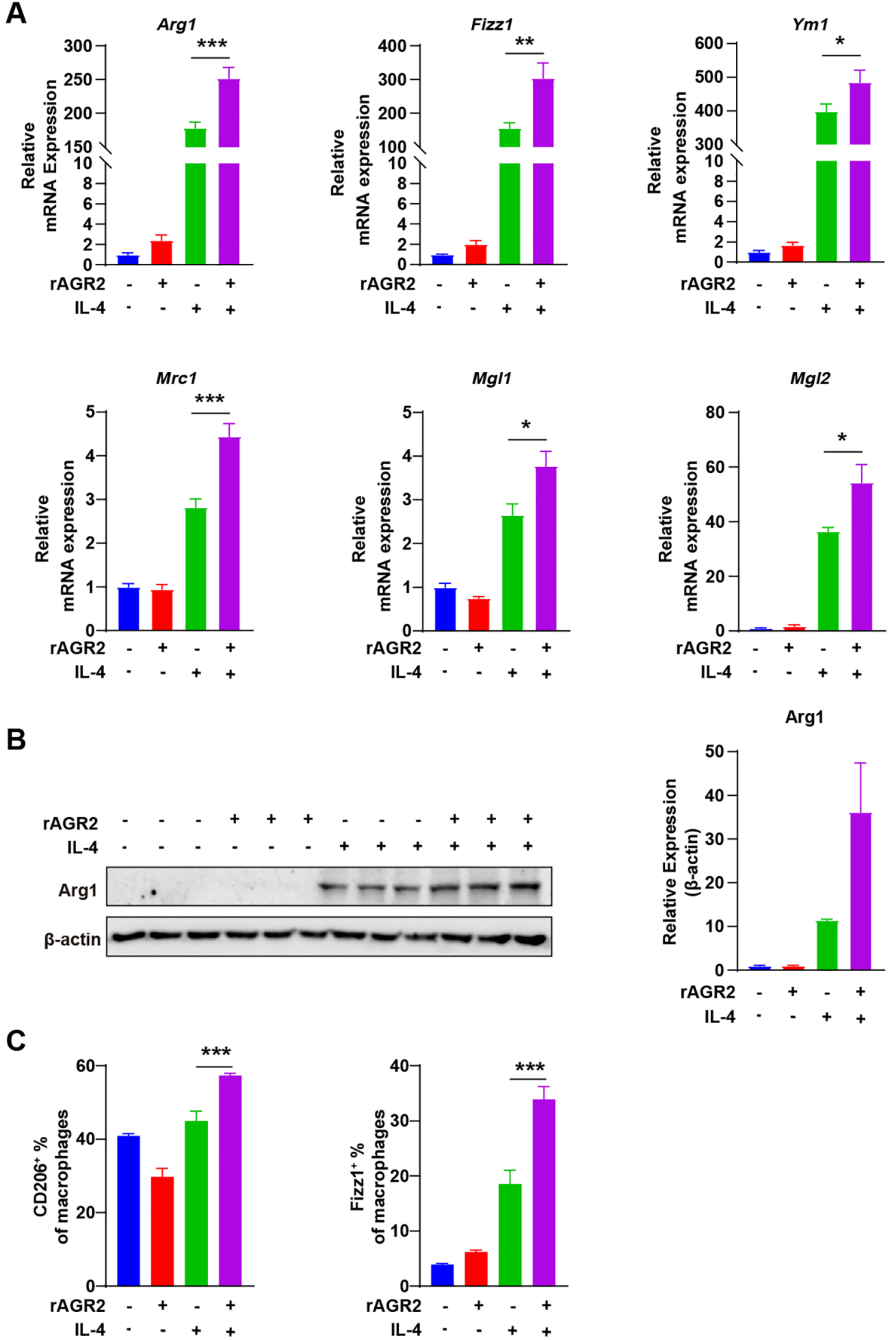
AGR2 promotes IL-4-induced M2 polarization of macrophages *in vitro.***(A–C)** Bone marrow-derived macrophages (BMDMs) were pre-treated with 100 ng/mL AGR2 and/or 20 ng/mL IL-4 for 24 hours during macrophage polarization. RT-PCR analysis of *Arg1*, *Fizz1*, *Ym1*, *Mrc1*, *Mgl1*, and *Mgl2* mRNA expression **(A)**. Left: Western blot analysis of Arg1 protein levels. Right: Quantification of Arg1 protein levels normalized to β-actin. Fold changes in Arg1 protein levels under AGR2 treatment relative to PBS (set to 1) were calculated **(B)**. Flow cytometric analysis showing the percentage of CD206^+^ macrophages and Fizz1^+^ macrophages **(C)**. *P < 0.05, **P < 0.01, and ***P < 0.001.

To investigate whether AGR2 modulates M2 polarization, we treated BMDMs with rAGR2 during IL-4-induced M2 differentiation. BMDMs were isolated and cultured *in vitro* as validated by morphological observation (**Supplementary Figure 1A**), and AGR2 expression in macrophages under different polarization states was first evaluated (**Supplementary Figure 1B**). RT-PCR analysis showed that IL-4 treatment for 24 h significantly upregulated the expression of M2 marker genes, including arginase 1 (*Arg1*), resistin-like alpha (*Fizz1*), chitinase 3-like 3 (*Ym1*), mannose receptor C type 1 (*Mrc1*), C-type lectin domain family 10 member A (*Mgl1*), and C-type lectin domain family 10 member B (*Mgl2*). Notably, co-treatment with rAGR2 further enhanced the expression of these M2-related genes (**Figure 3A**). Western blot analysis confirmed that rAGR2 alone did not induce Arg1 protein expression; however, co-treatment with IL-4 and rAGR2 significantly increased Arg1 protein levels compared with IL-4 treatment alone (**Figure 3B**). Flow cytometry analysis further demonstrated that rAGR2 upregulated the surface expression of CD206 and intracellular Fizz1 in IL-4-induced M2 macrophages (**Figure 3C**, **Supplementary Figures 2A, B**). These results collectively indicate that AGR2 effectively promotes IL-4-induced M2 polarization of macrophages *in vitro*.

### 3.4 AGR2 suppresses LPS-induced M1 polarization of macrophages *in vitro*

**Figure 4:**
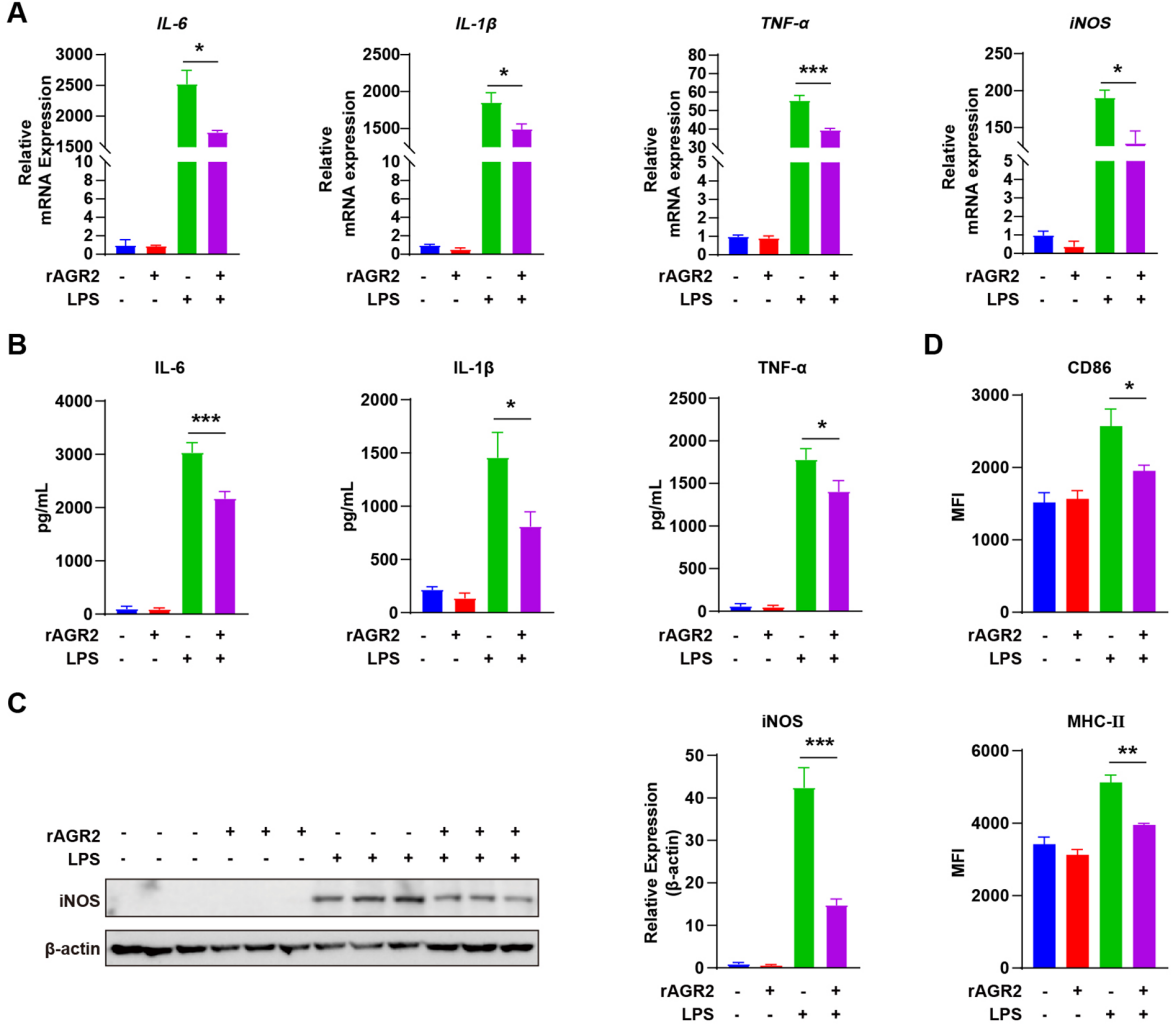
AGR2 suppresses LPS-induced M1 polarization of macrophages *in vitro.***(A–D)** BMDMs were pre-treated with 100 ng/mL AGR2 and/or 100 ng/mL LPS for 6 hours during macrophage polarization. RT-PCR analysis of *IL-6*, *IL-1β*, *TNF-α*, and *iNOS* mRNA expression **(A)**. ELISA analysis of IL-6, IL-1β, and TNF-α levels in cell culture supernatants **(B)**. Left: Western blot analysis of iNOS protein levels. Right: Quantification of iNOS protein levels normalized to β-actin. Fold changes in iNOS protein levels under AGR2 treatment relative to PBS (set to 1) were calculated **(C)**. Flow cytometric analysis showing the mean fluorescence intensity (MFI) of CD86 and MHC-II **(D)**. *P < 0.05, **P < 0.01, and ***P < 0.001.

M1 and M2 macrophages coexist in the TME, and their balance critically influences tumor progression. M1 macrophages exert anti-tumor effects by mediating anti-tumor immune responses (37, 38). To explore the effect of AGR2 on M1 polarization, we induced M1 differentiation in BMDMs using LPS and treated the cells with 100 ng/mL rAGR2—a concentration determined based on preliminary optimization experiments. RT-PCR analysis showed that rAGR2 treatment significantly reduced the expression of M1 marker genes, including IL-6, IL-1β, TNF-α, and iNOS (**Figure 4A**). ELISA of cell culture supernatants confirmed that rAGR2 decreased the secretion of IL-6, TNF-α, and IL-1β, consistent with the mRNA results (**Figure 4B**). Western blot analysis further validated that rAGR2 reduced iNOS protein expression (**Figure 4C**). Flow cytometry analysis revealed that rAGR2 decreased the expression of M1 activation markers CD86 and MHC-II in LPS-stimulated macrophages (**Figure 4D**, **Supplementary Figure 3**). These data demonstrate that AGR2 dynamically regulates macrophage polarization by promoting M2 differentiation and suppressing M1 polarization.

### 3.5 AGR2 enhances M2-conditioned medium-induced tumor cell migration, invasion, and proliferation *in vitro*

**Figure 5:**
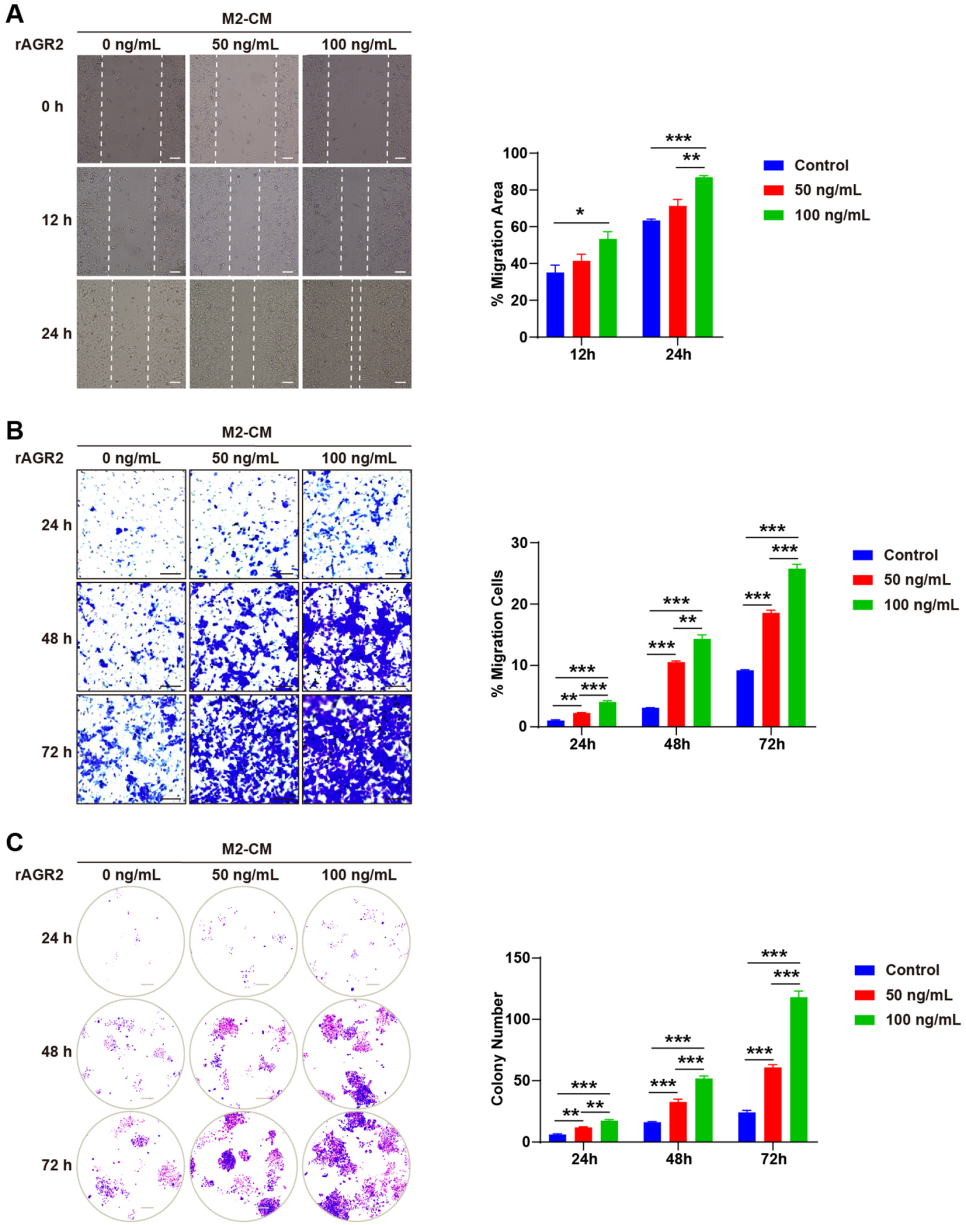
AGR2 enhances M2-conditioned medium-induced tumor cell migration, invasion, and proliferation *in vitro.***(A–C)** BMDMs were stimulated with 20 ng/mL IL-4 with or without 50 ng/mL or 100 ng/mL AGR2 for 48 hours, and the conditioned medium (CM) was collected. B16-F10 cells were cultured with different M2-CM. Migration ability of B16-F10 cells measured by wound healing assay. Scale bar: 100 μm **(A)**. Colony formation analysis of the effects of AGR2 on colony formation ability. Scale bar: 250 μm **(B)**. Number of invaded cells measured by transmigration assay **(C)**. *P < 0.05, **P < 0.01, and ***P < 0.001.

Given that AGR2 modulates macrophage polarization toward the immune-suppressive M2 phenotype, we hypothesized that this regulatory role contributes to its pro-tumorigenic effects. Since M2-polarized macrophages are well-documented to promote tumor migration, invasion, metastasis, angiogenesis, and cancer cell proliferation (39), we investigated whether AGR2 influences tumor cell functions via M2-derived factors using M2-CM. Wound healing assays showed that M2-CM significantly enhanced the migration of B16-F10 melanoma cells, and this effect was further amplified by rAGR2 in a concentration-dependent manner (**Figure 5A**). Transwell invasion assays demonstrated that M2-CM increased the invasive capacity of B16-F10 cells, and rAGR2 treatment further augmented this effect, with sustained enhancement observed up to 72 h (**Figure 5B**). Colony formation assays revealed that rAGR2 significantly increased the proliferation and colony-forming ability of B16-F10 cells cultured with M2-CM (**Figure 5C**). These findings indicate that AGR2 promotes M2-CM-induced tumor cell migration, invasion, and proliferation *in vitro*.

### 3.6 AGR2 requires CD98hc to activate the p-ERK/ERK signaling

**Figure 6:**
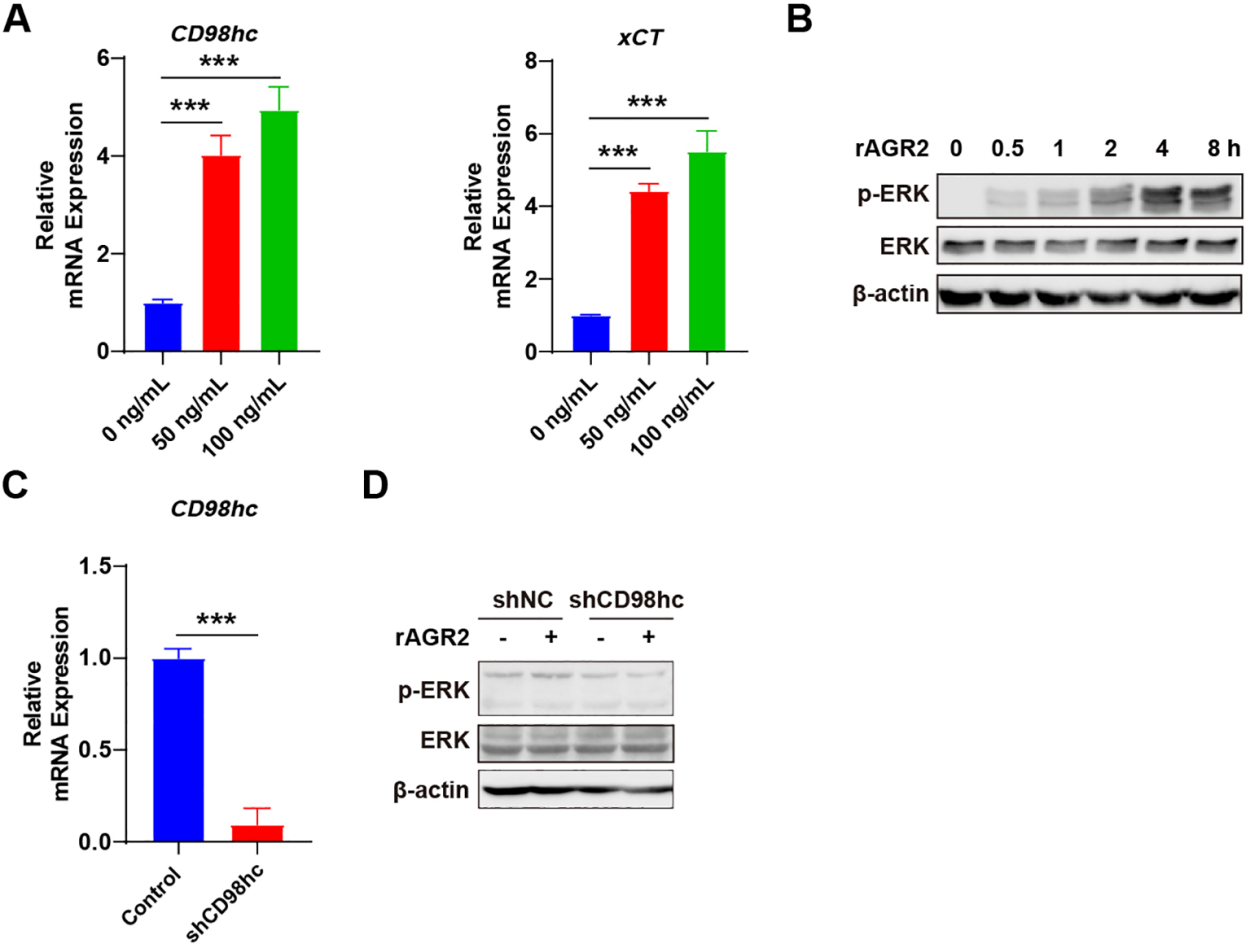
AGR2 requires CD98hc to activate the p-ERK/ERK signaling. **(A, B)** BMDMs were treated with 20 ng/mL IL-4 alone or in combination with 50 ng/mL or 100 ng/mL AGR2 for 48 h, and the corresponding CM was harvested. B16-F10 cells were then incubated with the different M2-CM. Relative mRNA expression levels of *CD98hc* and *xCT* in B16-F10 cells were detected via RT-PCR **(A)**. Protein levels of phosphorylated ERK (p-ERK) and total ERK in B16-F10 cells were analyzed by Western blot assay **(B)**. Efficiency of CD98hc knockdown by shCD98hc was verified by RT-PCR detection of *CD98hc* mRNA expression in B16-F10 cells **(C)**. Effects of CD98hc silencing on p-ERK and total ERK protein expression in B16-F10 cells were assessed using Western blot analysis **(D)**. ***P < 0.001.

To elucidate the molecular mechanism underlying AGR2-mediated tumor promotion, we first examined the expression of the AGR2 receptor complex CD98hc-xCT in tumor cells cultured with M2-CM. RT-PCR analysis showed that AGR2 treatment upregulated the expression of both *CD98hc* and *xCT* (**Figure 6A**). Given that previous studies have reported AGR2 binding to CD98hc-xCT induces ERK phosphorylation in neutrophils (19), we investigated the involvement of the ERK signaling pathway. Western blot analysis revealed that ERK phosphorylation (p-ERK) was detectable in macrophages as early as 30 min after rAGR2 stimulation, with p-ERK levels increasing in a time-dependent manner. Phosphorylation peaked at 4 h and remained elevated until 8 h, while total ERK protein levels remained unchanged (**Figure 6B**, **Supplementary Figure 4A**). The sustained activation of ERK over several hours suggests that AGR2 initiates a prolonged signaling cascade, which may be necessary for the stable transcriptional and functional reprogramming of macrophages toward the M2 phenotype. To confirm the role of CD98hc in AGR2-mediated ERK activation, we knocked down CD98hc expression using short hairpin RNA (shRNA) (**Figure 6C**). Notably, CD98hc silencing abrogated the AGR2-induced increase in p-ERK levels (**Figure 6D**, **Supplementary Figure 4B**). These results suggest that AGR2 requires CD98hc to activate the ERK signaling pathway. Based on prior research identifying CD98hc-xCT as an AGR2 receptor in other cell types (19), we propose that AGR2 likely engages with this receptor complex on macrophages to initiate downstream signaling, although direct binding in macrophages remains to be formally demonstrated.

### 3.7 AGR2 promotes tumor growth in tumor-bearing mice *in vivo*

**Figure 7:**
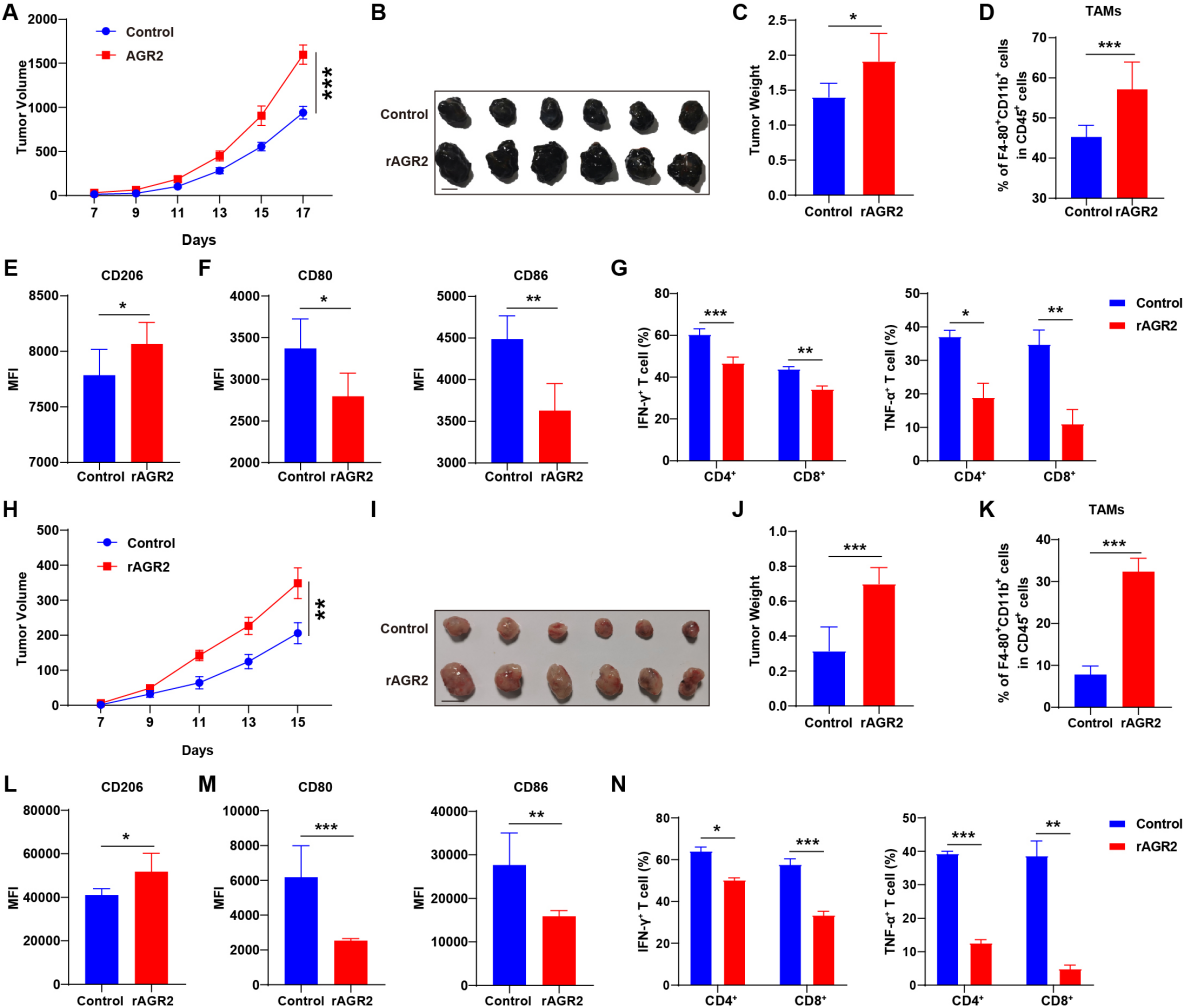
AGR2 promotes tumor growth in tumor-bearing mice *in vivo*. **(A–G)** Effects of AGR2 in a B16-F10 syngeneic mouse tumor model. Tumor growth curves **(A)**. Subcutaneous B16-F10 tumors were surgically removed and photographed. Scale bar: 1 cm **(B)**. Tumor weight at the endpoint **(C)**. Flow cytometric analysis of the percentage of tumor-associated macrophages (TAMs) in B16-F10 tumors at day 17 post-implantation **(D)**. **(E, F)** Flow cytometric analysis of MFI values for CD206 **(E)**, CD80, and CD86 **(F)** in TAMs. Flow cytometric detection of IFN-γ and TNF-α secretion levels in tumor-infiltrating CD4^+^ and CD8^+^ T cells **(G)**. **(H–N)** Effects of AGR2 in an LLC syngeneic mouse tumor model. Tumor growth curves **(H)**. Subcutaneous LLC tumors were surgically removed and photographed. Scale bar: 1 cm **(I)**. Tumor weight at the endpoint **(J)**. Flow cytometric analysis of the percentage of TAMs in LLC tumors at day 15 post-implantation **(K)**. **(L, M)** Flow cytometric analysis of MFI values for CD206 **(L)**, CD80, and CD86 **(M)** in TAMs. Flow cytometric detection of IFN-γ and TNF-α secretion levels in tumor-infiltrating CD4^+^ and CD8^+^ T cells **(N)**. *P < 0.05, **P < 0.01 and ***P < 0.001.

To validate our *in vitro* observations, we investigated whether AGR2 influences tumor growth and macrophage polarization *in vivo*. A subcutaneous mouse model was established by injecting B16-F10 cells into mice. Seven days post-injection, when tumors became palpable, intraperitoneal administration of AGR2 was initiated. Tumor growth was monitored by measuring tumor diameter and calculating tumor volume. In accordance with animal ethics guidelines, the experiment was terminated on day 17 in the B16-F10 xenograft melanoma model. We observed that AGR2 significantly increased tumor volume at the endpoint compared to the control group (**Figures 7A, B**). Additionally, excised tumor tissues from AGR2-treated mice exhibited a significant increase in tumor mass (**Figure 7C**).

To determine whether AGR2’s tumor-promoting effects are linked to macrophage polarization, we analyzed TAMs using flow cytometry. AGR2 treatment induced a notable increase in the accumulation of F4/80^+^CD11b^+^ TAMs (**Figure 7D**, **Supplementary Figure 5A**). Furthermore, AGR2 significantly enhanced the infiltration of CD206^+^ M2 macrophages (**Figure 7E**) while reducing the proportion and polarization of M1 macrophages, as indicated by decreased expression of CD80 and CD86 in the TME (**Figure 7F**). We further analyzed T cell function and found that rAGR2 treatment significantly suppressed the secretion of IFN-γ and TNF-α by CD4^+^ and CD8^+^ T cells (**Figure 7G**, **Supplementary Figures 5B** and **6A**).

To further confirm these findings, we established a second animal model by subcutaneously inoculating LLC cells. Consistent with the B16-F10 model, AGR2 treatment promoted tumor growth in the LLC lung cancer model, as evidenced by increased tumor volume and mass compared to the control group (**Figures 7H–J**). Flow cytometry analysis revealed that AGR2 significantly elevated the proportion of TAMs and the expression of the M2 marker CD206, while reducing the expression of M1 markers CD80 and CD86 (**Figures 7K–M**). Additionally, rAGR2 suppressed IFN-γ and TNF-α secretion by CD4^+^ and CD8^+^ T cells in the LLC model (**Figure 7N**, **Supplementary Figure 6B**). Collectively, these *in vivo* results demonstrate that AGR2 exerts pro-tumorigenic effects by promoting M2 macrophage polarization, suppressing M1 polarization, and inhibiting anti-tumor T cell function, thereby facilitating tumor growth.

## 4 Discussion

In this study, we demonstrated the key role of AGR2 in regulating macrophage polarization and tumor cell malignant behaviors. AGR2 orchestrates macrophage polarization toward the M2 pro-tumor subtype, as evidenced by the upregulated expression of M2 phenotypic markers. Meanwhile, AGR2 suppresses M1 macrophage polarization, leading to reduced expression of M1-associated pro-inflammatory cytokines and functional molecules. Our mechanistic data indicate that AGR2 requires CD98hc to activate the ERK signaling pathway. Mechanistically, AGR2 binds to the CD98hc-xCT receptor complex on the surface of tumor cells. This ligand-receptor interaction triggers the activation of the ERK signaling pathway, which in turn enhances the proliferative, invasive, and migratory capacities of tumor cells, ultimately facilitating tumor progression (**Supplementary Figure 7**).

A fundamental finding of our work is the differential expression pattern of AGR2 across 34 cancer types, with significant upregulation in 20 tumor types (including GBMLGG, LUAD, LIHC, and ESCA) compared to matched normal tissues. Critically, elevated AGR2 expression was tightly linked to poor prognosis in key malignancies such as LIHC, LUAD, and ESCA, as confirmed by Kaplan-Meier survival analysis. This aligns with and expands upon previous reports associating AGR2 overexpression with adverse outcomes in breast cancer and colorectal cancer (19, 27, 40), establishing AGR2 as a broadly applicable prognostic biomarker. Notably, our ESTIMATE algorithm analysis revealed a negative correlation between AGR2 expression and ESTIMATEScore, which reflects the combined abundance of stromal and immune components. AGR2-high tumors exhibited lower ESTIMATEScores, corresponding to reduced stromal and immune cell infiltration and increased tumor purity (calculated as 1- ESTIMATEScore). Further analysis confirmed that AGR2 expression was negatively correlated with both ImmuneScore and StromalScore. This observation provides a critical clinical context for AGR2’s functional role: its prognostic value is not merely a reflection of tumor cell intrinsic properties but is closely tied to its ability to remodel the TME. For cancers where AGR2 is downregulated (e.g., GBM, KIRC) or unchanged (e.g., LUSC, BLCA), further investigation is warranted to explore tissue-specific regulatory mechanisms, potentially involving alternative signaling cascades or microenvironmental cues that override AGR2’s oncogenic potential.

Macrophage plasticity is a defining feature of the TME, and our work identifies AGR2 as a key regulator of this process. We first demonstrated that AGR2 expression in TAMs correlates with tumor malignancy: in glioma, SKCM, and LUAD, AGR2 levels in TAMs progressively increased with advancing pathological stages. Immunohistochemical co-localization further pinpointed AGR2 expression in CD163-positive M2-like TAMs, directly linking AGR2 to the pro-tumor macrophage subtype. Functional *in vitro* studies confirmed this regulatory role: AGR2 not only promotes IL-4-induced M2 polarization but also suppresses LPS-induced M1 polarization. This dual regulation creates an immune-suppressive TME, as M1 macrophages—critical mediators of anti-tumor immunity (41–43)—are functionally impaired, while M2 macrophages—known drivers of tumor progression (44–46)—are amplified. We propose that macrophages themselves express and secrete AGR2, which may further enhance M2 polarization to form a potential positive feedback loop in the TME. However, this remains a speculative hypothesis that requires direct experimental validation in future studies. Our *in vivo* studies in B16-F10 melanoma and LLC lung cancer models validated these *in vitro* findings. AGR2 treatment significantly increased F4/80^+^CD11b^+^ TAM accumulation, elevated CD206^+^ M2 macrophage infiltration, and reduced M1 marker (CD80/CD86) expression. Concomitantly, AGR2 suppressed IFN-γ and TNF-α secretion by CD4^+^ and CD8^+^ T cells, directly impairing adaptive anti-tumor immunity. This multi-layered immune suppression—targeting both innate and adaptive compartments—explains the robust tumor-promoting effects of AGR2 observed *in vivo*, including increased tumor volume and mass in both xenograft models.

To dissect the molecular mechanism underlying AGR2’s effects, we focused on its interaction with the CD98hc-xCT receptor complex, a previously identified AGR2 binding partner in neutrophils (19). Our results show that AGR2 upregulates CD98hc and xCT expression in tumor cells and activates the p-ERK/ERK signaling pathway in a time-dependent manner. Critically, CD98hc knockdown abrogated AGR2-induced ERK phosphorylation, confirming the specificity of this signaling axis. This pathway directly contributes to tumor cell malignancy: AGR2-enhanced M2-CM significantly promoted tumor cell migration, invasion, and proliferation in a concentration-dependent manner. These findings integrate two key aspects of AGR2’s function: its paracrine regulation of macrophage polarization and its autocrine/paracrine activation of pro-tumor signaling in cancer cells. Notably, this mechanism is consistent with prior reports of AGR2 promoting colorectal cancer metastasis via CD98hc-xCT (19) and enhancing cell viability in pancreatic cancer via ERK-dependent c-Myc upregulation (47, 48), highlighting the conservation of this signaling axis across malignancies. However, a key limitation of our study is that we did not provide direct biochemical evidence (e.g., co-immunoprecipitation) for the physical interaction between AGR2 and CD98hc-xCT specifically in macrophages. It is possible that the signaling mechanism of AGR2 exhibits cell-type specificity, utilizing different receptor complexes or co-factors in different cellular contexts. Future studies are needed to definitively validate the AGR2-CD98hc-xCT interaction in macrophages and to explore potential alternative binding partners.

Our work builds upon the growing recognition of AGR2 as a secreted oncogenic factor by defining its role as a TME modulator (40, 49, 50). Previous studies have linked AGR2 to fibroblast infiltration and drug resistance (23, 24); our findings extend this to immune cell regulation, positioning AGR2 as a central node connecting stromal, immune, and tumor cell compartments. Regarding the relative contribution of different cell types to AGR2’s effects, our data collectively support a model in which the modulation of macrophage polarization is a central and primary mechanism. We demonstrated that AGR2 directly and potently skews macrophages toward an M2 phenotype while suppressing M1 polarization *in vitro*. The *in vivo* pro-tumor effects of AGR2 were closely associated with increased M2-like TAM infiltration and impaired anti-tumor T cell function. Although AGR2 can also activate the CD98hc/ERK pathway in tumor cells, this likely represents a secondary or synergistic axis that amplifies tumor malignancy, possibly in concert with factors secreted from AGR2-educated M2 macrophages. Thus, the rewiring of the tumor immune microenvironment via macrophage reprogramming appears to be the dominant pathway through which AGR2 exerts its tumor-promoting function.

In summary, our study demonstrates that AGR2 acts as a prognostic biomarker and key TME regulator in cancer. Its ability to promote M2 macrophage polarization, suppress M1 anti-tumor function, impair T cell immunity, and activate the CD98hc-xCT/ERK pathway to enhance tumor cell malignancy establishes AGR2 as a critical oncogenic factor. These findings provide a comprehensive framework for understanding AGR2’s role in cancer and support its potential as a therapeutic target for immunomodulatory cancer treatments.

## Data Availability

The original contributions presented in the study are included in the article/**Supplementary Material**. Further inquiries can be directed to the corresponding authors.
