# Supplementary material for: AGR2 promotes tumor progression by regulating macrophage polarization via the CD98hc-xCT/p-ERK pathway
Source: Front Immunol. 2026 Feb 26;17:1774238. doi: 10.3389/fimmu.2026.1774238 (PMC12979147; doi:10.3389/fimmu.2026.1774238)
Supplement: Supplementary file 1 [file Table1.docx]

**Supplementary Material Table of Contents**

Supplementary Figure 1 .....................................................................................page 2

Supplementary Figure 2 .....................................................................................page 3

Supplementary Figure 3 .....................................................................................page 4

Supplementary Figure 4 .....................................................................................page 5

Supplementary Figure 5 .....................................................................................page 6

Supplementary Figure 6 .....................................................................................page 7

Supplementary Figure 7 .....................................................................................page 8

Supplementary Table 1 .......................................................................................page 9

Supplementary Table 2 .......................................................................................page 10

**Supplementary Figure 1**

**AGR2 is expressed in macrophages.** (A) Flow cytometry analysis to determine the purity of BMDMs. (B) Representative confocal microscopy images showing AGR2 (red) expression in M0, M1, and M2 macrophages. Nuclei were stained with DAPI (blue). Scale bar: 10 µm.

**
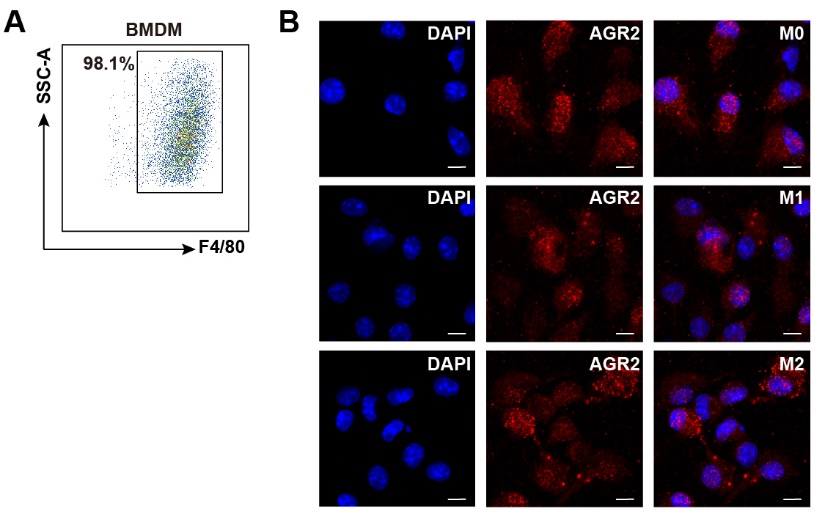
**

**Supplementary Figure 2**

**AGR2 promotes M2 polarization of macrophages *in vitro.*** Flow cytometric analysis showing representative images of the percentage of CD206⁺ macrophages (A) and Fizz1⁺ macrophages (B).


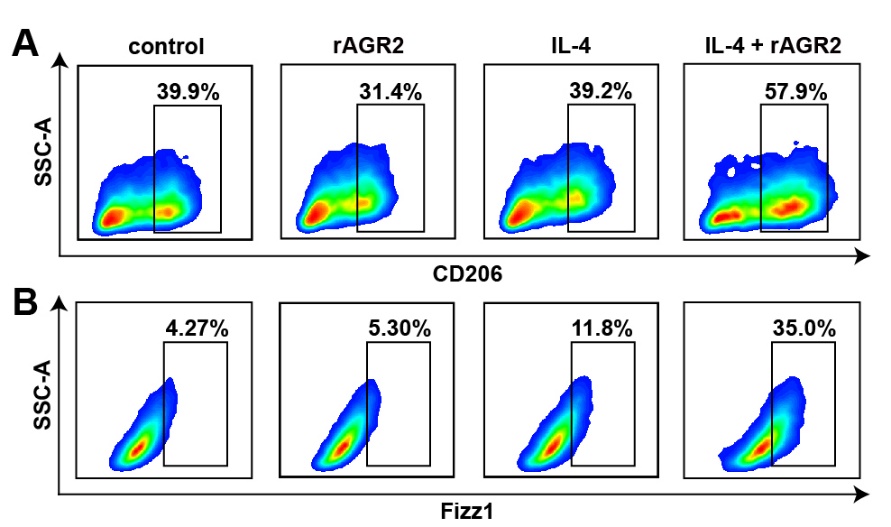


**Supplementary Figure 3**

**AGR2 suppresses LPS-induced M1 polarization of macrophages *in vitro.*** Flow cytometric analysis showing representative images of the MFI of CD86 and MHC-II.


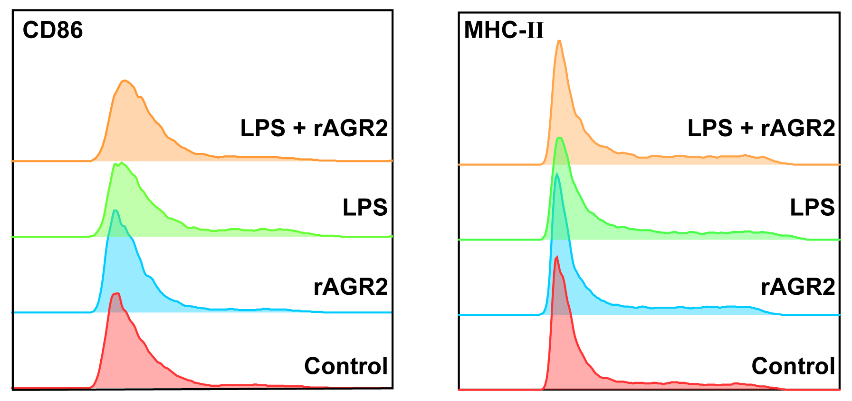


**Supplementary Figure 4**

**AGR2 requires CD98hc to activate the p-ERK/ERK signaling.** Quantification of p-ERK/ERK ratio. Fold changes in p-ERK levels upon AGR2 treatment and CD98hc silencing were normalized to PBS (set as 1, A). Fold changes in p-ERK levels upon AGR2 treatment were normalized to PBS (set as 1, B).


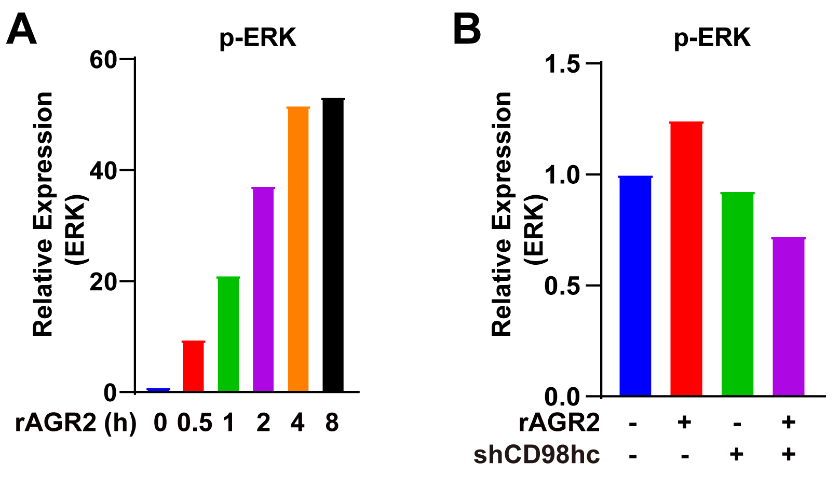


**Supplementary Figure 5**

**Gating strategy.** Flow cytometry gating strategy to identify TAMs from the total TIIs (A). Flow cytometry gating scheme to detect and quantify the secretion levels of IFN-γ and TNF-α in T cells sorted from the total TIIs (B).

**
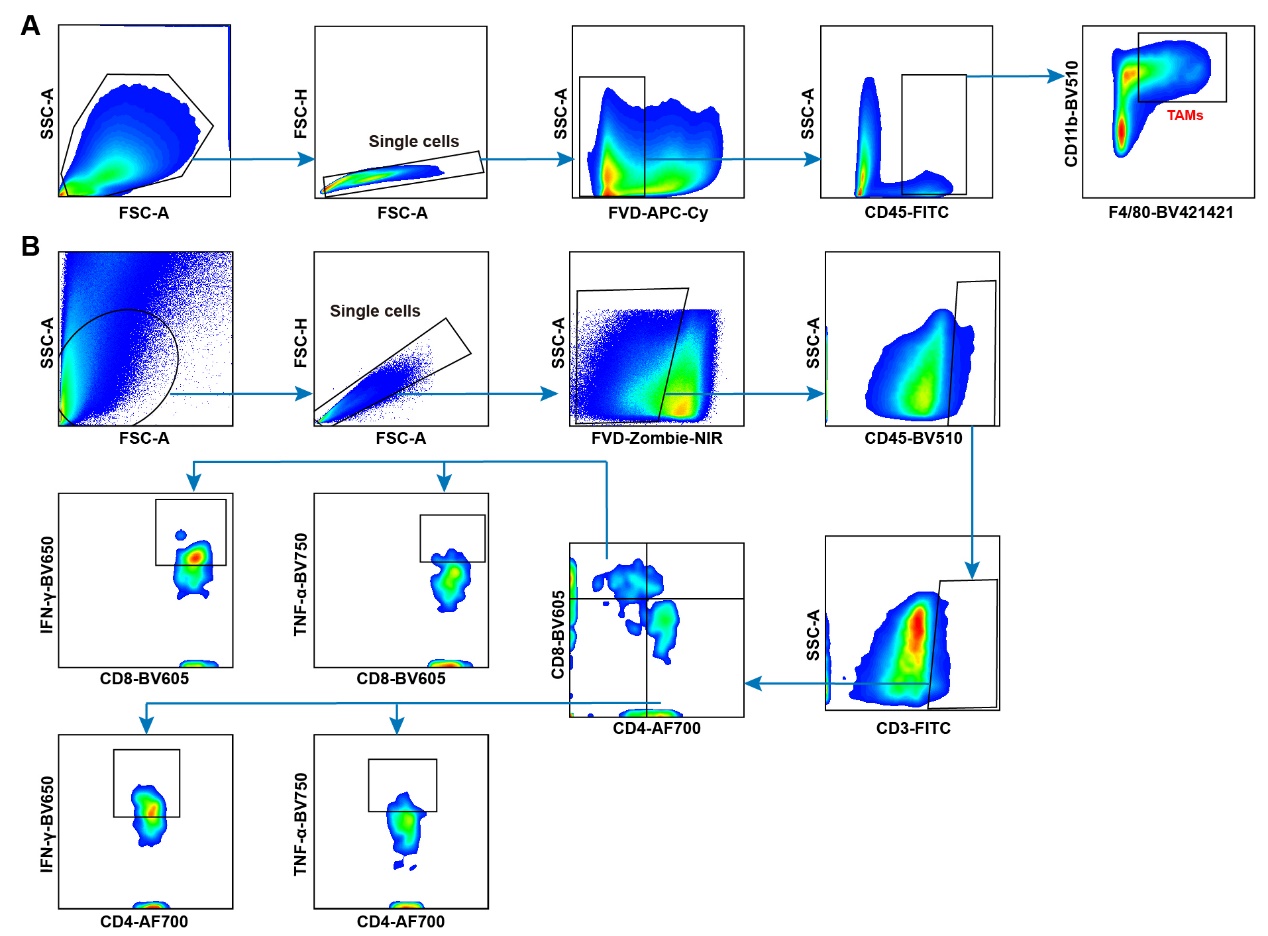
**

**Supplementary Figure 6**

**AGR2 promotes tumor growth in tumor-bearing mice *in vivo*.** Representative flow cytometric plots depicting the secretion of IFN-γ and TNF-α by tumor-infiltrating CD4⁺ and CD8⁺ T cells in B16-F10 (A) and LLC (B) xenograft models.


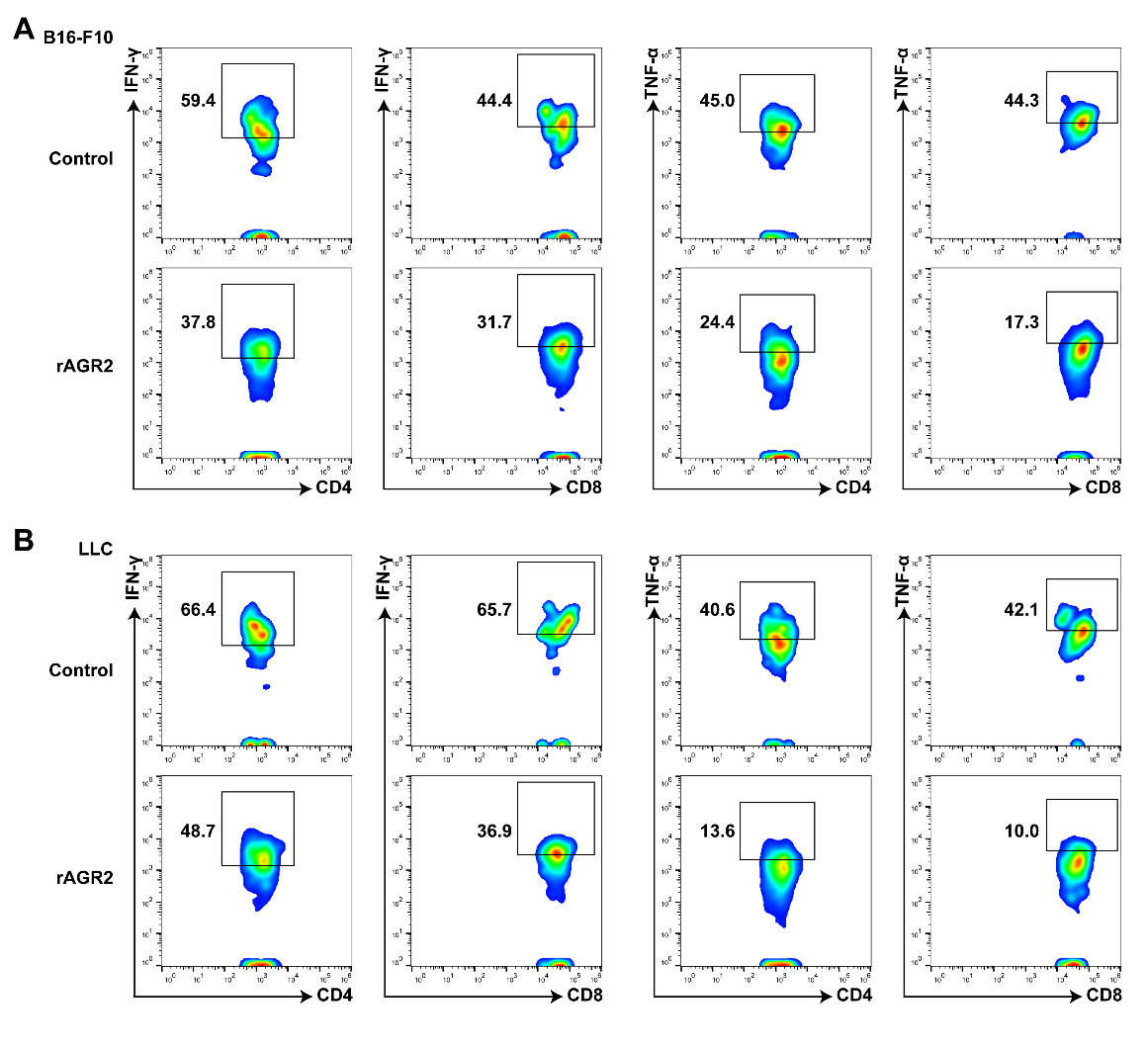


**Supplementary Figure 7**

**Schematic of AGR2-mediated tumor-promoting mechanism.** Schematic summary of the key role of AGR2 in regulating macrophage polarization and tumor cell malignant behaviors. AGR2 orchestrates macrophage polarization toward the M2 pro-tumor subtype, as evidenced by the upregulated expression of M2 phenotypic markers (Arg1, CD206, Fizz1). Meanwhile, AGR2 suppresses M1 macrophage polarization, leading to reduced expression of M1-associated pro-inflammatory cytokines and functional molecules (IL-6, IL-1β, iNOS). Mechanistically, AGR2 binds to the CD98hc-xCT receptor complex on the surface of tumor cells. This ligand-receptor interaction triggers the activation of the ERK signaling pathway, which in turn enhances the proliferative, invasive, and migratory capacities of tumor cells, ultimately facilitating tumor progression.


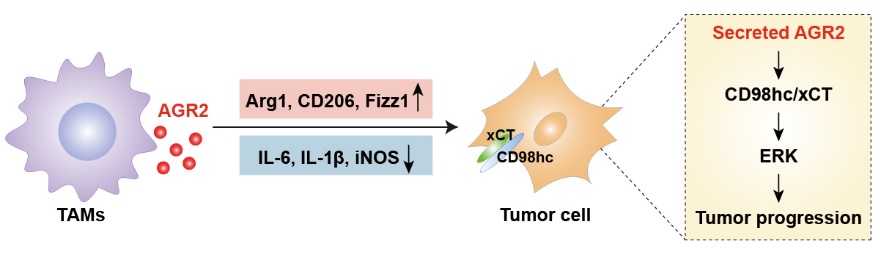


**Supplementary Table 1**

**Antibodies.**

| **Antibodies** | **Source** | **Identifier** |
| --- | --- | --- |
| Anti-Anterior Gradient 2 antibody | Abcam | CAT# ab76473 |
| Anti-CD68 antibody | Abcam | CAT# ab283654 |
| Anti-CD163 antibody | Abcam | CAT# ab182422 |
| anti-ERK antibody | Cell Signaling Technology | CAT# 4695S |
| anti-pERK antibody | Cell Signaling Technology | CAT# 4370S |
| Arginase-1 Polyclonal antibody | Proteintech | CAT# 16001-1-AP |
| NOS2 (M-19) | Santa Cruz | CAT# sc-650 |
| Beta Actin Monoclonal antibody | Proteintech | CAT# 66009-1-Ig |
| Anti-mouse CD16/32 Antibody | Biolegend | CAT# 101320 |
| FITC anti-mouse CD45 Antibody | Biolegend | CAT# 157214 |
| Brilliant Violet 510 anti-mouse CD45 Antibody | Biolegend | CAT# 103138 |
| Brilliant Violet 510™ anti-mouse/human CD11b Antibody | Biolegend | CAT# 101245 |
| Brilliant Violet 421™ anti-mouse F4/80 Antibody | Biolegend | CAT# 123132 |
| PE anti-mouse CD206 (MMR) Antibody | Biolegend | CAT# 141706 |
| PE/Cyanine5 anti-mouse CD80 Antibody | Biolegend | CAT# 104711 |
| APC anti-mouse CD86 Antibody | Biolegend | CAT# 105012 |
| Alexa Fluor® 488 anti-mouse I-A/I-E | Biolegend | CAT# 107616 |
| FITC anti-mouse CD3 Antibody | Biolegend | CAT# 100204 |
| Alexa Fluor 700 anti-mouse CD4 Antibody | Biolegend | CAT# 100430 |
| Brilliant Violet 605 anti-mouse CD8a Antibody | Biolegend | CAT# 100743 |
| Brilliant Violet 650 anti-mouse IFN-γ Antibody | Biolegend | CAT# 505832 |
| Brilliant Violet 750 anti-mouse TNF-α Antibody | Biolegend | CAT# 506358 |

**Supplementary Table 2**

**Primers used for RT-PCR.**

| **Gene** | **Forward Primer 5’-3’** | **Reverse Primer 5’-3’** |
| --- | --- | --- |
| *β-actin* | CCGTAGATGAAAGGCAAACTCT | ATGGAGCCACCGATCCACA |
| *Arg1* | CCACAGTCTGGCAGTTGGAAG | GGTTGTCAGGGGAGTGTTGATG |
| *Fizz1* | CCTGCTGGGATGACTGCTA | TGGGTTCTCCACCTCTTCAT |
| *Ym1* | GCCACTGAGGTCTGGGATGC | TCCTTGAGCCACTGAGCCTTC |
| *Mrc1* | AAGGCTATCCTGGTGGAAGAA | AGGGAAGGGTCAGTCTGTGTT |
| *Mgl1* | CAGAATCGCTTAGCCAATGTGG | TCCCAGTCCGTGTCCGAAC |
| *Mgl2* | TTCAAGAATTGGAGGCCACT | CAGACATCGTCATTCCAACG |
| *IL-6* | CCCAATTTCCAATGCTCTCCTA | AGGAATGTCCACAAACTGATATGCT |
| *IL-1β* | AAGGAGAACCAAGCAACGACAAAATA | TTTCCATCTTCTTCTTTGGGTATTGC |
| *TNF-α* | ACGGCATGGATCTCAAAGAC | AGATAGCAAATCGGCTGACG |
| *iNOS* | ATCTTTGCCACCAAGATGGCCTGG | TTCCTGTGCTGTGCTACAGTTCCG |
| *CD98hc* | TGCTGCTGCTACTGGCTTTC | TTGCTGGTCTTGAAGGTGGT |
| *xCT* | GGTGGTGTGTTTGCTGTC | GGTGGTGTGTTTGCTGTC |
